# Impact of Yoga as an Add-On Intervention on Neurocognitive Functions Among Adult Athletes: A Pilot Study

**DOI:** 10.7759/cureus.44797

**Published:** 2023-09-06

**Authors:** Naveen G Halappa, Kamlesh Jha, Vijayabanu U, Harishankar Singh

**Affiliations:** 1 Department of Yoga, School of Yoga, Naturopathy and Cognitive Studies, Babasaheb Bhimrao Ambedkar University, Lucknow, IND; 2 Department of Physiology, All India Institute of Medical Sciences, Patna, IND; 3 Department of Psychology, School of Social Sciences and Languages, Vellore Institute of Technology, Chennai, IND

**Keywords:** cognition, exercise, athletes, sports, yoga

## Abstract

Background: Athletes are vulnerable to musculoskeletal injuries and psychiatric conditions. Previous studies have reported the benefits of yoga on cognitive functions among healthy children, adults, and the elderly. This pilot study aimed to test the neurocognitive functions among athletes with/without yoga intervention.

Methods: A non-randomized, two-armed parallel-group, single-blind comparative trial was used. The participants were grouped into (i) yoga with sports activity (YSA, n = 15) and (ii) sports activity alone (SA, n = 14). The subjects were assessed at the baseline and after a one-month intervention using digit span forward (DSF), digit span backward (DSB), Trail Making Test (TMT) A & B, and Rey Auditory Verbal Learning Test (RAVLT). A comprehensive one-hour yoga training three days a week for two months constitutes selected asanas (postures), pranayama (breathing techniques), relaxation techniques, and meditation techniques. The control group constitutes the routine sports activity for the same period.

Results: A paired sample t-test showed a significant improvement in cognitive performance on TMT A & B duration and RAVLT total score in the YSA group compared with the SA group. However, a significant trend was observed for DSF, DSB, and RAVLT immediate recall. Independent sample t-test (pre-post change scores) showed no significant group difference in cognitive performance, except there was a significant trend observed related to DSF (p = 0.053) and RAVLT distraction (p = 0.09), where the yoga group showed better performance in cognitive functions.

Conclusion: The results suggest that yoga may be integrated with sports to enhance neurocognitive functions.

## Introduction

Athletes are vulnerable to musculoskeletal injuries. Such injuries have a strong emotional impact, leading to a spectrum of neuropsychiatric disturbances and musculoskeletal disorders. The most common conditions are strains, sprains, back pain, anxiety, depression, and eating disorders [1-5]. Regular moderate exercise has been shown to reduce inflammation, reduce the activity of sympathetic nervous system functions, and oxidative stress [6-9]. However, high-intensity, unaccustomed sports activity/exercise, and overtraining can damage muscle and connective tissues with infiltration of inflammatory cells [10].

Evidence suggests that those involved in sports in late childhood are less prone to cognitive decline and emotional issues [11]. To substantiate this, one study reported cognitive flexibility was associated with better performance and low anxiety and stress levels among athletes [12].

Yoga is beneficial in the management of stress [13]. Also, yoga therapy can have an effect on improving cognitive functions, which is one of the measures of neuroplasticity, such as working memory, attention, concentration, and executive functions among healthy and depressed individuals [14,15].

Although several studies have been conducted to test the effect of yoga on neuropsychological functions among healthy volunteers and few medical conditions, none of the studies has been done to test the neurocognitive functions in athletes with/without yoga intervention. Therefore, a pilot study was needed to test the effect of a comprehensive yoga module as an add-on intervention on neurocognitive functions among adult athletes.

## Materials and methods

Study design

The present study was a non-randomized, two-armed parallel-group, single-blind comparative trial conducted between July 2017 and March 2018. The participants were grouped into (i) yoga with sports activity (YSA, n = 22) and (ii) sports activity alone (SA, n = 16). The age, sex, and education of each study participant were matched with the control group. The duly filled informed consent was taken after explaining the research design to the participants. The study was approved by the Ethical Committee of Leibniz University, Hannover, Germany (EV LUH 08/2017).

Participants

**Figure 1:**
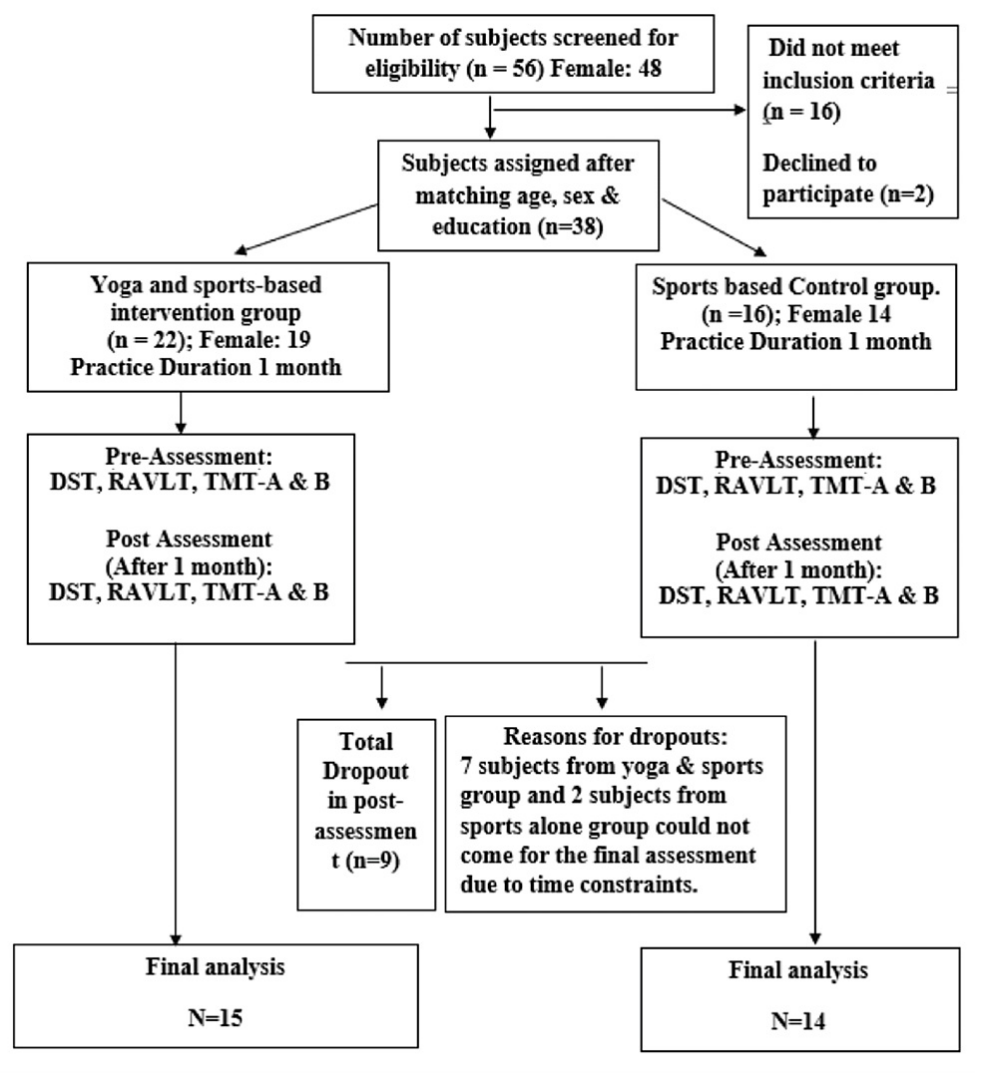
CONSORT flow chart of the study CONSORT: Consolidated Standards of Reporting Trials; DST: digit span test; TMT: Trail Making Test; RAVLT: Rey Auditory Verbal Learning Test.

The professionals involved in sports were invited to participate in this study through electronic communication. Thirty-eight participants of both genders from a public university in Germany who engaged in any sports activity were recruited in this study. Of these, eight participants (two from the control/sports alone group) could not complete the post-assessment (Figure 1).

The study was conducted at the Institute of Sports Science of the university. No incentive was given to the participants to take part in this study. The healthy subjects were screened based on the General Health Questionnaire (GHQ), aged between 18 and 60 years, English-known subjects, and subjects with a minimum primary school education of seven years who could read English letters and numerals. The participants were the students, faculty, and staff working at the Leibniz University Hannover and also the general public in and around the city of Hannover, Germany who were involved in any sports activity for a minimum of one year. However, participants with known medical conditions and participants who received yoga training for the past three months were excluded from the study. Fifty-six participants were screened after making electronic communication to participate in the study. Among those, 38 were eligible for the study after applying inclusion and exclusion criteria.

Yoga intervention

The participants in the yoga group attended comprehensive yoga module training for three days a week for two months. The participants were encouraged to practice yoga at home on the remaining four days a week upon completion of at least one week. Each session of yoga practice lasted for one hour. This consisted of selected asanas (postures), pranayama (breathing techniques), relaxation techniques, and meditation techniques. Visual demonstration before each yoga practice, verbal guidance, and hands-on assistance were given to correct the method. Besides this, a weekly half-hour health talk on the principles of yoga therapy and its scientific/therapeutical benefits was included in yoga education. The details of the yoga intervention are given in Table 1. The participants were asked to report any untoward event during the study period. Yoga was taught by a registered yoga and naturopathy medical practitioner (five-year medical degree program) from India who also had a post-graduation degree and a Ph.D. in yoga. He has more than 10 years of professional experience in Yoga. Yoga class attendance was monitored by another professional.

**Table 1:** Yoga intervention

S. No.	Yoga practice type	Duration
1	Sukshma vyayama (light exercises)	5 min
2	Surya namaskar (Sun salutation) - 4 rounds	6 min
3	Shavasana (corpse pose)	2 min
4	Padahastasana (hand to foot pose)	2 min
5	Ardha chakrasana (half wheel pose)	2 min
6	Vakrasana (half spinal twist pose)	2 min
7	Ardha ushtrasana (half camel pose)	2 min
8	Bhujangasana (cobra pose)	2 min
9	Pavanamuktasana (wind relieving pose)	2 min
10	Sarvangasana (shoulder stand pose)	2 min
11	Sethubandasana (bridge pose)	2 min
12	Shavasana (corpse pose)	2 min
13	Kapalbhati pranayama - 5 rounds	3 min
14	Nadishodhana pranayama - 9 rounds	5 min
15	Ujjayi pranayama - 6 rounds	3 min
16	Brahmari pranayama - 9 rounds	3 min
17	Aum meditation	15 min
	Total	60 min

Salient Features of the Yoga Training Protocol in a Phased Manner

Week one: Introduction to rules and regulations of yoga practice (before, during, and after the yoga practice) followed by an introduction to yoga postures and relaxation techniques. The asanas were taught following traditional Patanjali's emphasis on mindfulness.

Week two: Introduction of suryanamskara, kapalabhati kriya, and pranayama with week one practices.

Week three: Introduction of meditation technique with week one and week two practices.

Weeks four to nine: Continuation of complete yoga module.

Sports activity

The participants in the sports activity alone/control group attended their routine sports activity for a minimum of three days a week during a two-month study period.

Assessments

The demographic and sports experience data including age, gender, education, occupation, and previous sports experience were collected using a structured questionnaire.

Neurocognitive functions assessment was done at baseline and after two months. The researcher who assessed the neurocognitive functions was blind to the group status. The test included the following: (i) Rey Auditory Verbal Learning Test (RAVLT): verbal learning, immediate memory span, delayed recall, recognition, new learning, and susceptibility for interference can be assessed with this [16]. (ii) Digit span test (DST): attention and immediate recall (short-term memory) can be assessed with a digit span forward, whereas working memory can be evaluated with a digit span backward [17]. (iii) Trail Making Test (TMT) A & B involves motor speed and attention functions. The TMT provides information on visual search, scanning, processing speed, mental flexibility, and executive functions [18].

Statistical analysis

The data have been tested for normality of distribution using the Shapiro-Wilk test. Statistical software SPSS version 20 (IBM Corp., Armonk, NY) has been used for statistical analysis. Independent sample and chi-square tests were used to assess the baseline differences between the two groups concerning socio-demographic details, sports experience, and neurocognitive functions. A paired-sample t-test was used to assess within-group differences concerning neurocognitive functions. An independent sample t-test was used to analyze the change from baseline to one month between two groups (pre-post change scores). The effect size was calculated for paired sample test significant value. The sample size was calculated using G*Power software, the two-tailed, larger effect size of 0.8, power of 0.8, and a significance level of 0.05, which resulted from 26 subjects in each group.

## Results

Nine participants dropped out of the study for post-assessment due to time constraints. Finally, 15 subjects in the YSA and 14 in the SA groups were available for post-assessment (Figure 1).

Baseline comparison

Table 2 presents the baseline comparison of yoga with sports and sports alone group concerning sociodemographic details, total sports experience with a duration of practice, attrition rate, and neurocognitive functions observed between YSA and SA groups. There was no significant difference observed between the two groups. However, a significant baseline difference was observed related to the total number of hours spent in sports (p = 0.01). Results suggest that the sports alone group spent more duration in sports than the yoga and sports group.

**Table 2:** Sociodemographic, sports experience with duration, attrition, and neurocognitive function characteristics at the baseline * P < 0.05 is considered as significant. # Categorical variables - chi-square test. RAVLT: Rey Auditory Verbal Learning Test. # Continuous variables - independent samples t-test. ^ Expressed as number (percentage as whole %).

Parameter	Yoga with sports activity (n = 22), study	Sports activity alone (n = 16), control	t/χ^2^	p
Mean (SD) age in years	29.54 (8.50)	29.75 (8.22)	-0.74	0.941
Female#^	19	14	0.010	0.919
Mean (SD) education in years	17.59 (1.33)	16.87 (1.02)	1.79	0.081
Occupation#^			5.74	0.057*
Faculty/staff	4 (18.18%)	4 (25%)		
Students	16 (72.72%)	6 (37.5%)		
General public	2 (9.09%)	6 (37.5%)		
Total sports experience in years	9.68 (13.05)	14.00 (11.88)	-1.045	0.303
Total number of hours spent in sports	1299.81 (1577.72)	2805.37 (2177.48)	-2.475	0.01*
Attrition	7 (27.3%)	2 (12.5%)	1.216	0.270
Neurocognitive functions^#^				
Digit span forward	5.00 (1.23)	4.88 (1.02)	0.330	0.743
Digit span backward	3.55 (1.14)	3.38 (0.88)	0.497	0.622
Trail-making duration ‘A’ (seconds)	30.59 (8.68)	29.81 (7.11)	0.294	0.771
Trail-making error ‘A’	0.23 (0.42)	0.38 (0.50)	-0.978	0.335
Trail-making duration ‘B’ (seconds)	65.57 (17.12)	56.94 (9.52)	1.811	0.079
Trail-making error ‘B’	0.50 (0.88)	0.44 (0.51)	0.250	0.804
RAVLT total score	60.63 (10.36)	61.93 (5.77)	-0.448	0.657
RAVLT distraction list B	8.26 (2.40)	8.25 (2.01)	0.017	0.986
RAVLT immediate recall	12.58 (2.91)	12.44 (2.15)	0.161	0.873

Within-group analysis

Table 3 presents within-group comparisons in YSA and SA groups using paired sample t-tests. There was a significant improvement in cognitive performance on TMT A & B duration and RAVLT total score in the YSA group compared to the SA group except for RAVLT immediate recall (p = 0.02). Further, a significant trend was observed for digit span forward (DSF), digit span backward (DSB), and RAVLT immediate recall.

**Table 3:** Changes in yoga with sports activity and sports activity alone groups ^a^ Values are denoted as mean (standard deviation). ^b^ Paired sample t-test for within-group comparison. ^c^ Independent sample t-test for the changes in difference scores between the groups. * P < 0.05 is considered as significant. RAVLT: Rey Auditory Verbal Learning Test.

Variable		Participants (n = 29), mean (standard deviation)^a^		Baseline to two-month change^b^		Between-group (change score)^c^	
	Group	Baseline	After two months	t	p	t	p
Digit span forward	Yoga with sports	5.20 (1.37)	5.67 (1.11)	-1.825^b^	0.089	-2.026	0.053
	Sports	4.93 (1.07)	4.71 (0.72)	1.00^b^	0.336		
Digit span backward	Yoga with sports	3.47 (1.24)	3.87 (1.187)	-2.103^b^	0.054	-1.014	0.320
	Sports	3.36 (0.92)	3.43 (0.85)	-0.268^b^	0.793		
Trail-making duration ‘A’ (seconds)	Yoga with sports	30.20 (9.12)	26.20 (6.316)	2.169^b^	0.048*	1.025	0.314
	Sports	29.71 (7.63)	28.43 (5.90)	0.677^b^	0.510		
Trail-making error ‘A’	Yoga with sports	0.13 (0.35)	0.27 (0.59)	-0.695^b^	0.499	-0.976	0.338
	Sports	0.36 (0.49)	0.29 (0.61)	0.291^b^	0.775		
Trail-making duration ‘B’ (seconds)	Yoga with sports	65.53 (17.90)	48.93 (13.21)	4.420^b^	0.001*	1.317	0.199
	Sports	56.86 (8.97)	54.64 (12.04)	0.825^b ^	0.424		
Trail-making error ‘B’	Yoga with sports	0.40 (.828)	0.13 (0.35)	1.169^b^	0.262	0.335	0.740
	Sports	0.57 (0.64)	0.43 (0.75)	0.486^b^	0.635		
RAVLT total score	Yoga with sports	59.66 (10.67)	66.73 (5.81)	-2.967	0.01*	-1.094	0.284
	Sports	62.35 (5.87)	66.00 (5.94)	-1.825	0.091		
RAVLT distraction list B	Yoga with sports	8.13 (2.44)	8.80 (1.97)	-1.022	0.324	-1.760	0.090
	Sports	8.00 (2.00)	7.00 (2.11)	1.455	0.169		
RAVLT immediate recall	Yoga with sports	12.20 (3.05)	13.53 (1.84)	-1.848	0.086	-0.401	0.692
	Sports	12.64 (1.59)	13.64 (1.82)	-2.6460	0.020*		

Between-group analysis

Table 3 also presents between-group comparisons in YSA and SA groups using independent sample t-tests (pre-post change scores). There was no significant group difference in cognitive performance, except a significant trend related to DSF (p = 0.053) and RAVLT distraction (p = 0.09), where the yoga group had a better performance in cognitive functions.

## Discussion

The main aim of this pilot study was to test the neurocognitive functions among athletes with/without yoga intervention. In this study, we found a significant improvement in cognitive performance on TMT A & B duration and RAVLT total score in the YSA group compared to the SA group except for RAVLT immediate recall (p = 0.02). Further, a significant trend was observed for DSF, DSB, and RAVLT immediate recall. The effect size for TMT A & B duration is 0.56 and 0.48, respectively, indicating a medium effect size. The results indicate better attention, immediate recall (short-term memory), working memory, verbal learning, motor speed, mental flexibility, and executive functions in the yoga group. Also, there was no significant group difference in cognitive performance, except a significant trend related to DSF and RAVLT distraction. The yoga group showed better performance in cognitive functions indicating the yoga group showed better performance than the sports group related to attention with reduced distraction.

The findings of this pilot study on improvement in neurocognitive functions with yoga practice are consistent with previous studies that tested the impact of yoga on children, adolescents, and the elderly [19-21]. To substantiate this, a systematic review and meta-analysis of yoga on cognition in the elderly reported a moderate effect size on attention, memory, and executive functions [19].

Plausible mechanisms behind enhancing neurocognitive functions in yoga and sports groups might be changes in the brain-derived neurotrophic factor (BDNF) levels, gamma-aminobutyric acid (GABA), and reduction in cortisol levels [14,22]. Also, meditation practice has improved structural and functional neuroplasticity by influencing brain regions such as the prefrontal cortex (PFC) and right anterior insula, responsible for attention, sensory processing, and interoception [23,24]. Further, meditation practice resulted in GABAergic cortical inhibition, which is implicated in better emotional regulation and cognition [25]. Another plausible mechanism responsible, especially the meditation component of yoga, is the association of the default mode network (DMN) with the cognitive functions of an individual [26].

Strengths

This is the first study to test the neuropsychological functions in the sports activity population with/without yoga intervention. The rater who assessed neurocognitive functions was blind to the group status, another strength of the study. Also, note that the CONSORT extension for nonpharmacological trials and the CLARIFY guidelines have been followed to report this study [27,28].

Limitation

The study's main limitation is the lack of randomization and low sample size, which may have resulted in restricted generalizability and type II errors. Another major limitation is that RAVLT delayed recall and recognition were not tested due to time constraints that need rectification in future studies. Furthermore, the confounding effect of variation in sports activity across the group cannot be ruled out.

## Conclusions

This pilot study provides initial evidence concerning the benefits of yoga as an add-on intervention with sports on neurocognitive function performance, one of the neuroplasticity measures. The evidence suggests that yoga may be integrated with sports and into sports curricula to enhance neurocognitive functions and sports performance.

Future randomized controlled studies should focus on including other neurocognitive tests with larger sample sizes and also to check the plasma BDNF levels, which is one of the measures of neuroplastic changes in the brain. Further, correlating these changes with brain wave patterns using an electroencephalogram.
